# Shedding Light on Metals Release from Chestnut Wood to Wine Spirit Using ICP-MS

**DOI:** 10.3390/foods11223617

**Published:** 2022-11-12

**Authors:** Sofia Catarino, Vasiliki Thanasi, Gael Morin, Ofélia Anjos, Tiago A. Fernandes, Ilda Caldeira, Laurent Fargeton, Benjamin Boissier, Sara Canas

**Affiliations:** 1 LEAF—Linking Landscape, Environment, Agriculture and Food—Research Center, Associated Laboratory TERRA, Instituto Superior de Agronomia, Universidade de Lisboa, Tapada da Ajuda, 1349-017 Lisboa, Portugal; vasilikithanasi@isa.ulisboa.pt (V.T.); gael.morin@agrosupdijon.fr (G.M.); 2 CEFEMA—Center of Physics and Engineering of Advanced Materials, Instituto Superior Técnico, Universidade de Lisboa, Av. Rovisco Pais, 1, 1049-001 Lisboa, Portugal; 3 Instituto Politécnico de Castelo Branco, Quinta da Senhora de Mércules, 6001-909 Castelo Branco, Portugal; ofelia@ipcbpt; 4 CEF—Centro de Estudos Florestais, Instituto Superior de Agronomia, Universidade de Lisboa, Tapada da Ajuda, 1349-017 Lisboa, Portugal; 5 CQE—Centro de Química Estrutural, Institute of Molecular Sciences, Departamento de Engenharia Química, Instituto Superior Técnico, Universidade de Lisboa, Av. Rovisco Pais, 1049-001 Lisboa, Portugal; tiago.a.fernandes@tecnico.ulisboa.pt; 6 DCeT—Departamento de Ciências e Tecnologia, Universidade Aberta, Rua da Escola Politécnica,141-147, 1269-001 Lisboa, Portugal; 7 Instituto Nacional de Investigação Agrária e Veterinária, Quinta de Almoínha, Polo de Dois Portos, 2565-191 Dois Portos, Portugal; ilda.caldeira@iniav.pt (I.C.); sara.canas@iniav.pt (S.C.); 8 MED—Mediterranean Institute for Agriculture, Environment and Development & CHANGE—Global Change and Sustainability Institute, Instituto de Investigação e Formação Avançada, Universidade de Évora, Pólo da Mitra, Ap. 94, 7006-554 Évora, Portugal; 9 Vivelys, Domaine du Chapître, 34750 Villeneuve-les-Maguelone, France; laurent.fargeton@vivelys.com (L.F.); benjamin.boissier@vivelys.com (B.B.)

**Keywords:** wine spirit, mineral composition, heavy metals, ageing, chestnut wood, ICP-MS

## Abstract

Possible effects caused by mineral elements during wine spirit ageing are diverse. In this study, the evolution of the mineral composition of wine spirit during ageing with chestnut (*Castanea sativa* Mill.) wood was investigated. A wine distillate was aged in 250 L wooden barrels (traditional ageing) and in 50 L glass demijohns with wood staves and micro-oxygenation (alternative ageing). Sampling was performed after 21, 60, 180, 270, and 365 days of ageing. The elemental composition of the wine spirits, including alkaline, alkaline earth metals, and heavy metals, was assessed by quadrupole inductively coupled plasma mass spectrometry (Q-ICP-MS). For most of the elements, no significant differences between wine spirits from distinct ageing modalities were observed. Ageing time had significant effect on most of them, with different trends and distinct magnitude of changes, depending on each specific element. The concentrations of the mineral elements found in the wine spirits were very low, especially those of heavy metals, which is quite positive in terms of quality and food safety. Novel information on metals released from chestnut wood to wine spirits confirms its appropriateness for ageing this beverage.

## 1. Introduction

The mineral content of wine spirit (WS) is of particular importance due to its potential influence on physicochemical stability, sensory characteristics, and food safety [1]. Calcium (Ca) and iron (Fe) can form insoluble compounds, causing an increase of WS turbidity [2]. Transition metals, such as Fe and copper (Cu), seem to act as catalysts in redox reactions in WSs involving phenolic compounds and other substrates, such as ethanol [3]. In particular, Fe leads to the positive colour evolution of distillates to yellow or brown hues [4,5]. In distillates characterized by increased Cu content and some sulphurous acid content, a reddish deposit of copper-sulphide is formed under the action of light [4]. Some elements can remove unwanted odours—Cu stands out for its role in the treatment of sulphidic off-odours [6,7]—while others can be used as authentication markers [8,9,10,11,12,13,14,15]. As trace elements, some metals, such as manganese (Mn), Fe, cobalt (Co), nickel (Ni), Cu, zinc (Zn), and molybdenum (Mo), are essential for human health, but their accumulation can be adverse [16]. Other trace elements such as arsenic (As), cadmium (Cd), and lead (Pb) are of high concern because of their toxicological and/or carcinogenic properties [17]. Furthermore, the presence of high metal contents imparts bitterness and astringency to the distillates. In particular, Al and Zn, apart from Fe and Cu, can generate a bitter taste [4]. 

A series of mineral elements, including alkaline, alkaline earth elements, and several heavy metals, have been quantified in spirit beverages—though at extremely low concentrations [3,5,11,18,19,20,21,22]—but only limited data is available for WSs. 

Transference of mineral elements from wine to WSs during the distillation process is not expected (as metals are not volatile); however, some elements can be introduced during this production step by the contact with metallic surfaces [23]. Traditionally, column stills and alembics are made of Cu or contain Cu inner surfaces, and can thus release Cu to a significant extent [23]. Other potential sources of mineral enrichment during the production of WSs are related to materials and equipment used during storage, ageing, blending, and filtration steps, and with the quality of the water used to dilute the aged WSs [1,5].

**Figure 1:**
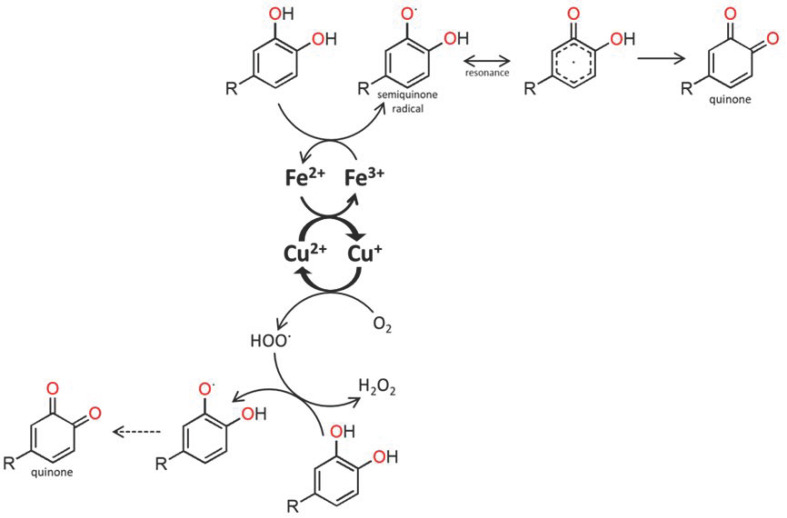
Proposed scheme depicting the catalytic activity of iron/copper ions in the nonenzymatic catechols’ oxidation for the production of quinones and hydrogen peroxide.

The role of some transition metals, e.g., Mn, Fe, and Cu, as catalysts of oxidation reactions in wine has been deeply examined [24,25,26,27,28,29]; thus, it was foreseen that similar behaviour may occur in WSs, when considered as a reaction vessel. This is due to their ability to readily redox cycle (readily donate and accept electrons), reducing molecular oxygen to reactive oxygen species (e.g., hydroxyl radicals, (HO˙)) [27,28], capable of oxidizing almost any organic molecule. Of particular relevance are the reactions involving phenolic compounds with a catechol or a galloyl group, which are sequentially oxidized to semiquinones and quinones, while oxygen (O_2_) is reduced, in turn, to hydroperoxyl radicals (HO_2_˙) and hydrogen peroxide (H_2_O_2_). This process is mediated by redox cycling of Fe^3+^/Fe^2+^ and Cu^2+^/Cu^+^ couples, as shown by Figure 1 [25,26,28].

The content of these transition metals seems to affect specific oxidation mechanisms involving substrates for oxidation in WSs, namely, phenolic compounds extracted or derived from the wood, and ethanol [19]. In fact, the effect of Cu content on specific oxidation mechanisms in sugarcane spirits has been observed [19]. Recently, the involvement of this metal as a catalyst in hydrogen cyanide and ethanol reaction, giving rise to ethyl carbamate, in sugarcane spirits, was reported [30]. 

WSs are traditionally aged in oak wooden barrels (mainly from *Quercus robur* L. species) to enhance their final quality [31,32]. During wooden ageing, many physicochemical reactions occur, affecting the quality characteristics and organoleptic profile of the WS. These reactions involve oxygen, which is continuously supplied through the micropores of the wood and the stave gaps, many components of the WS itself, and of the wood (low molecular weight compounds, tannins, and mineral elements) [33]. Although it results in high quality WS, this is a time-consuming and costly technology, among other drawbacks. On those grounds, in the last years, alternative ageing technologies have been explored, namely, the addition of wood fragments to the spirits kept in stainless steel vats, in order to meet sustainability criteria and accelerate the ageing process [31,34,35]. In the recent past, the use of wood fragments combined with micro-oxygenation (MOX), to mimic what occurs in a wooden barrel, demonstrated that is possible to accelerate the ageing process, compared with that of barrel ageing. In addition, chestnut wood (*Castanea sativa* Mill.) has been introduced as an alternative option [36]. Bearing in mind that K, Ca, Mg, Na, and Fe, are among the main inorganic components of wood ash [37], the potential release of these metals to WSs during ageing is foreseen [3,38,39,40], especially as the wood often undergoes a thermal treatment (toasting) during the barrel-making process. Furthermore, the transference of mineral elements from wood to WS seems to depend on the botanical species, as shown in a recent and interesting study on the transference of wood-extractable elements in spirit model solution, involving different wood species, including oak, both *Quercus robur* and *Quercus petraea* [40].

Several studies have been performed to investigate the effect of wooden ageing on the chemical composition, sensory attributes, and colour evolution of WSs [32,41,42,43,44,45,46]. However, only scarce data are available regarding the effect of wood on the elemental composition of WSs and other spirits during the ageing step and, except for our recent publication focused on Fe and Cu elements [3], no data is available for chestnut wood. 

Within the European Union, the composition of WS is controlled [47], but the mineral element content is not. Despite significant consumption of WS, its inorganic profile remains poorly defined and further research is needed. Understanding the evolution of mineral composition throughout WS production, and how it is influenced by the different technological stages, is fundamental to preserve their high quality and safety. 

In this context, the main aim of this study, developed under Project Oxyrebrand (https://projects.iniav.pt/oxyrebrand/index.php/pt/, accessed on 15 August 2022), was to examine the effect of WS’s ageing with chestnut wood—considering both traditional (wooden barrel) and alternative technologies (staves combined with different MOX levels)—on the beverage’s mineral composition, namely, alkaline, alkaline earth elements, and several heavy metals. In addition, the evolution of mineral composition during 365 days of ageing was investigated. To our best knowledge, no such research was disclosed before. 

## 2. Materials and Methods

### 2.1. Experimental Design and Wine Spirit Samples

**Figure 2:**
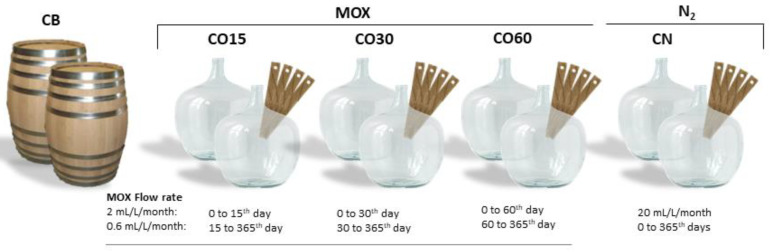
Scheme of the ageing experiment.

A wine distillate was aged during 365 days in 250 L wooden barrels (traditional ageing, CB) and in 50 L glass demijohns using wood staves and MOX (alternative ageing system), with two independent replicates (Figure 2), as previously described by the authors [45]. Regarding the alternative system, three MOX modalities (CO15, CO30, and CO60) and one control modality with nitrogen application (CN) were considered. Both barrels and demijohns were placed in the cellar of Adega Cooperativa da Lourinhã (Lourinhã, Portugal) under similar environmental conditions.

Portuguese chestnut wood (*Castanea sativa* Mill.) was used both in the barrels and in the staves. J. M. Gonçalves cooperage (Palaçoulo, Portugal) manufactured the staves (50 cm length, 5 cm width, and 1.8 cm thickness) with medium plus toasting level (90 min at an average temperature of 240 °C; 1.8 cm toasting thickness). The staves were treated in an industrial oven and the barrels were heated over a fire of wood offcuts, under controlled temperature, to assure a similar toasting level. The number of staves inserted into the demijohns was determined to replicate the surface area to volume ratio (85 cm^2^/L) of a 250 L barrel. 

The general physicochemical characteristics of the wine distillate were as follows: alcoholic strength, 78.30 vol.; pH, 5.33; total acidity, as acetic acid, 0.12 g/L of absolute ethanol; volatile acidity, as acetic acid, 0.09 g/L of absolute ethanol.

MOX was applied to the WS in the glass demijohns during the ageing period, supplying pure oxygen (X50S Food, Gasin, Portugal), as previously described [3]. Flow rates were selected based on the results of our previous study [48]. In addition, pure nitrogen (X50S Food, Gasin, Portugal) was applied continuously over the ageing time through a specific device (Gasin, Portugal) to the WS in one of the ageing modalities (N) in order to decrease the dissolved oxygen as much as possible, acting as a control [3].

**Figure 3:**
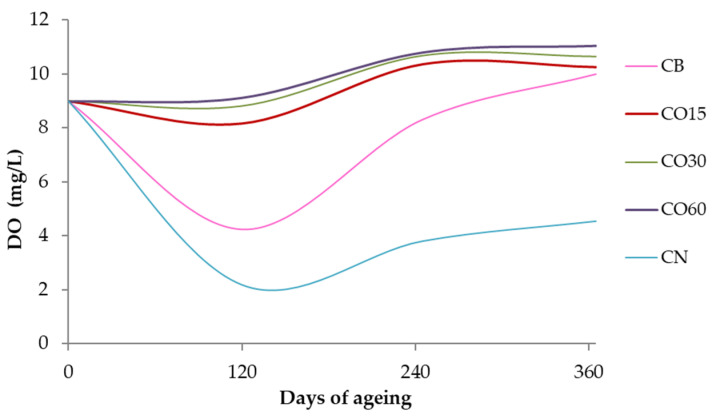
Average dissolved oxygen concentrations in the WSs, during the ageing period [45].

The dissolved oxygen content in each modality was controlled over time [3], and its levels are shown in Figure 3.

The aged WSs were collected at 21, 60, 180, 270, and 365 days, representing a total of 51 samples for elemental analysis, including the wine distillate.

### 2.2. Multi-Elemental Analysis of WSs by ICP-MS

Before the analysis by ICP-MS, the WSs were centrifuged at 5000 g for 10 min (Heraeus Biofuge Stratos Centrifuge, Thermo Fisher Scientific, Waltham, Massachusetts, USA, and concentrated (from 35 to 15 mL) using a SpeedVac concentrator (Labconco, model 7810033, Kansas City, MO, USA at 65 ± 1 °C for 120 min, in order to reduce the ethanol content. While ICP-MS offers high sensitivity and excellent detection limits for many elements, interferences on key elements arising from the carbon content can be problematic, thus requiring sample preparation [49]. Thereafter, the volume was made up to the initial volume (35 mL) with ultrapure water.

Multi-elemental analysis was carried out with an Elan 9000 ICP-MS (Perkin-Elmer SCIEX, Norwalk, CT, USA) equipped as described by Catarino et al. [50]. The operating conditions of the ICP-MS equipment were as follows: radio-frequency (RF) power of 1200 W; Ar gas flow rates of 15 L/min for cooling, between 0.94 and 0.98 L/min for nebulizer, and 1.5 L/min for auxiliary; solution uptake rate of 1.0 mL/min.

Element concentrations were determined in WS samples (treated as previously described) and after 10-fold dilution, in duplicate, by adapting the ICP-MS semi-quantitative method described by Catarino et al. [50]. In this manner, the final ethanol content in the samples, in the blank solutions, and in the standard solutions used for analytical calibration was similar. A full mass spectrum (m/z = 6 − 240, omitting the mass ranges 16–18; 40, 41, 211–229) was obtained by full mass range scanning. Rhodium (Rh) and rhenium (Re) (10 µg/L) were used as internal standards, in order to offset non-spectroscopic interferences, such as matrix-induced suppression and instrumental bias.

The reference response table (Perkin-Elmer TotalQuant III, Perkin-Elmer SCIEX, Norwalk, CT, USA) was updated using different multi-elemental standard solutions with appropriate concentrations for WS analyses. The equipment sampling system was rinsed with 2% HNO_3_ (*v/v*) for 75 s between determinations.

To avoid contamination, all polyethylene material (volumetric flasks, micropipette tips, and autosampler vessels) was immersed for at least 24 h in 20% (*v/v*) HNO_3_ and rinsed thoroughly with purified water before use. For decontamination solution preparation, reagent grade HNO_3_ was double-distilled using an infrared sub-boiling distillatory system (model BSB-939-IR, Berghof, Germany). Purified water (conductivity < 0.1 µS/cm) was produced using an Arium Comfort apparatus (Sartorius, Göttingen, Germany).

### 2.3. Statistical Analysis

Bearing in mind the objectives and the experimental design of this study, ANOVA was chosen for statistical treatment of the data. Indeed, a one-way analysis of variance (ANOVA) was carried out to assess the effect of the ageing modality (B, O15, O30, O60, and N), as a fixed factor, on the mineral element content of the aged WSs in each sampling time. Another one-way ANOVA was performed to examine the significance of mineral elements kinetics over the ageing time. Fisher’s least significant difference (LSD) test was applied to compare the averages when a significant difference (*p* < 0.05) was detected. In addition, heat map was used to analyse the correlations between the minerals and the modalities for 360 days of ageing. All the calculations were carried out using Statistica version 7.0 software (Statsoft Inc., Tulsa, OK, USA).

## 3. Results

### 3.1. Mineral Elements Concentration during Ageing Experiment

The present study aimed a better understanding of the influence of wood ageing, comparing the traditional technology (wooden barrel) and an alternative technology (staves combined with different micro-oxygenation levels), in both cases using chestnut wood, on the mineral element content of WS, over time. As previously described, enrichments can occur during the WS ageing process, by contact with metallic surfaces and due to mineral release from the wood. Bearing in mind that K, Ca, Mg, Na, and Fe, among other elements, are the main components of wood ash, their transference to WS during ageing is expected. The wine distillate itself (non-aged WS) presents some mineral elements introduced during the distillation step, although mineral transference from wine to WS during distillation is not expected. Increased concentration of minerals can also result from the addition of water to the aged WS, which is a regular practice to decrease the alcoholic strength before bottling. However, this practice was not performed in the WSs resulting from this ageing essay, which should be taken into account in the interpretation and discussion of the results. It should be emphasized that for all the elements monitored in this study, the concentrations found in the wine distillate and in the aged WSs were quite low or even below the detection limits of the analytical method applied. Numerous elements, namely, Be, B, Ca, Sc, Ti, V, Ge, Se, Y, Zr, Nb, Sn, Sb, Cs, Ba, Pr, Nd, Sm, Eu, Gd, Tb, Dy, Ho, Er, Tm, Yb, Lu, Hf, Ta, W, Pt, Hg, Tl, Bi, Th, and U, were absent (below the detection limit of the analytical method) in the wine distillate, and most of them were also not detected in all the aged WSs. The concentrations of Li, Na, Mg, K, Ca, Rb, and Sr (alkaline and alkaline earth elements), and of Al, Mn, Co, Ni, Zn, As, Cd, Mo, and Pb (most of them heavy metals), and the ANOVA results are displayed in Table 1 and Table 2, together with the associated graphics, to clearly highlight the significant level of the changes observed and to better explain the behaviours of these mineral elements according to the ageing modalities (CB, CO15, CO30, CO60, and CN) and the ageing time (21, 60, 180, 270, and 365 days). In addition, the wine distillate was analysed at the beginning of the experiment; the results (0 days) are shown in the same Tables. The contents of Fe and Cu (not included in this study), assessed by flame atomic absorption spectrophotometry, have been disclosed in a recent publication by the authors in the scope of Project Oxyrebrand [3], with regard to their role as catalysts of oxidation reactions. The information on the ageing conditions (Figure 2) and on the levels of dissolved oxygen assessed in the different ageing modalities during the experiment (Figure 3) are of great interest to understand the behaviour observed during the ageing period. In fact, depending on the redox environment, metal ions can exist under different oxidation forms at different proportions, thus influencing their solubility and toxicity. In addition, as expected, the evolution of pH during the experiment, from 5.33 in the wine distillate to values close to 4.20 in all the WSs at 365 days, can be helpful for a better interpretation of the results.

#### 3.1.1. Solubility Properties to Consider

In a context as complex as that of a WS’s reaction medium, ageing in contact with wood, in which the effect of continuous extraction of wood constituents, in addition to the colligative properties must be considered (boiling point, depression or freezing point, and osmotic pressure), some empirical solubility rules should be used to predict the products of precipitation reactions and, therefore, to understand the results obtained: Metal oxides (O^2−^) are typically insoluble compounds, with the exception of alkaline and alkaline earth elements. Carbonates (CO_3_^2−^), oxalates (C_2_O_4_^2−^), and hydroxides (OH^−^) all give rise to soluble compounds with the metals under discussion. Acetates (CH_3_CO_2_^−^), chlorides (Cl^−^), or bromides (Br^−^) can, on the other hand, be generated in the WS reaction medium, and are generally soluble in water and ethanol. In general, the solubility of the Group 2 elements that form compounds with single-charged negative ions (e.g., OH^−^) increases as the period increases. As a result, Mg(OH)_2_ is less soluble than Sr(OH)_2_. Compounds containing doubly-charged negative ions (e.g., SO_4_^2−^ or CO_3_^2−^) reduce solubility as the group decreases. MgSO_4_ is thus more soluble than SrSO_4_ [51].

Compounds possessing ions of widely varying radii are frequently soluble in water, according to a general rule that is commonly followed. Salts with limited water solubility, on the other hand, are ions with comparable radii. In general, the size difference favors water solubility. Empirically, an ionic compound MX is more soluble when the radius of M^+^ is about 0.8 Å smaller than that of X^−^. Nevertheless, the species that may form with studied metals of the analysed WSs include not only halogens, nitrates, chlorates or perchlorates, sulphates or sulphides, carbonates, oxalates, oxides or hydroxides, but also acetates, tartrates, and other weak acid species. The solubility of a precipitate comprising an anion with basic properties, or a cation with acidic properties, or both, is pH dependent. The persistence of these minerals in solution will certainly be determined by competition and the ability to generate soluble species, as well as the nature and amount of the WS solutes and solvents present, the kind of wood, and the temperature and pressure to which the system is subjected [51].

#### 3.1.2. Alkaline and Alkaline Earth Elements

The one-way ANOVA results for the contents of the alkaline and alkaline earth elements Li, Na, Mg, K, Ca, Rb, and Sr in the WSs during the 365 days of ageing are shown in Table 1. In general, for all these elements and ageing modalites, enrichments were observed, indicating mineral release from wood to the distillate, which may have been favoured by the decrease of pH over time [52]. Among this group of metals, Ca presents particular interest owing to its potential participation in physicochemical instability phenomena, causing haze to the beverage [53]. From a different point of view, Li, Rb, and, to a low extent, Mg and Sr, have been pointed out as indicators of the geographic origin of wine, as they can be easily absorbed by plants in the soil [54]. However, as the transference of mineral elements from wine to WS during distillation is unlikely, geographical fingerprinting techniques based on multi-element composition of WS will reflect mainly anthropogenic factors [54]. In spite of that, some studies on authentication of spirits based on this approach have been published [14].

The concentrations of Li found in the WSs were quite low, varying between 1.4 µg/L (wine distillate) and 4.4 µg/L (CO15, 270 days) (Table 1). At each sampling time, the Li content of the WSs was not significantly influenced by the ageing modality. For the ageing modalities CO15 and CO30, significant increases were observed in concentration by time, until 270 days of ageing. This enrichment, although not relevant from the technological point of view, is most probably explained by Li release from the wood into the WSs throughout ageing. In fact, in a recent study on the release of wood extractable elements in an experimental spirit model (wood sawdust was extracted with 60% *v/v* ethanol solution, during seven days under stirring), involving different wood species (other than chestnut) from distinct geographic origins, the transference of Li to the spirit model solutions was observed [40]. From 270 to 365 days, a significant decrease was found in these WSs. A similar behaviour was observed in the other ageing modalities, even without a significant effect of time (most probably explained by the high variation between replicates). Comparing with other spirits, much higher concentrations, ranging from 4 to 1260 µg/L, were reported for sugarcane spirit (cachaça) [5].

Regarding Na, the concentrations were very low in all the WSs, ranging from 698 µg/L (wine distillate) to 1459 µg/L (CB, 365 days) (Table 1). After 21 days of ageing, the WS aged in barrel (CB) exhibited the highest content in Na (611 µg/L), followed by CO15 and CO60, and, finally, by the CN and CO30 modalities. However, at the other sampling times, no significant differences between WSs were observed, though CB was always associated with the highest levels. For WSs corresponding to CO15, CO30, and CN modalities, significant decreases were observed in concentration until 21 days of ageing. Between 21 and 60 days of ageing, steady concentrations were noticed in all the WSs. The increase of Na, not relevant from the technological point of view, occurred especially between 180 and 270 days, which can be ascribed to its transference from the wood; this hypothesis is supported by the aformentioned research [40]. After 270 days, different behaviour was shown, according to each modality. The concentrations found were similar to those observed in ethanolic extracts (spirit model) obtained from different kinds of wood, namely, oak, cherry, plum, and mulberry [40], though the results cannot be directly compared, and were lower than those reported for Sherry and Penedés spirits [8], tequila and mezcal [11], scotch whiskey [10], and grape marc distillates [43]. However, as previously stressed, the literature mainly reports studies developed with spirits purchased in the market, already diluted with water. Finally, Na together with Mg, Ca, and Sr were the most abundant elements found in the WSs at the end of the ageing essay.

Mg is essential for the proper functioning of the human body [16]. No significant differences were found in Mg content between the WSs resulting from distinct ageing technologies (Table 1). As expected, the wine distillate showed the lowest concentration (128 µg/L), while the highest level was exhibited at the end of the experiment by the WS submitted to an intermediate level of oxygen (CO30) (1544 µg/L). Regardless of the ageing modality, progressive increases in Mg content were observed throughout the ageing essay, agreeing with previous works [38,40]. This finding, combined with the fact that magnesium salts are soluble [53], thus unlikely to precipitate, suggests the continous extraction of this mineral element from the wood to the WS. According to the literature, the WSs seem to have similar levels to those reported for Sherry and Penedés spirits [8], tequila and mezcal [11], whisk(e)y [12], Cognac, rum [10], grape marc distillates [43], and sugarcane spirits (cachaça) [55].

The ageing modality had no significant effect on the K content of the WSs at any sampling time (Table 1). However, at 180, 270, and 365 days of ageing, higher content of this alkaline metal (420-524 µg/L) was found in the WS aged in barrel (CB). Lower (144-206 µg/L) K contents, although of the same order of magnitude, were exhibited by the WSs submitted to the lowest level of MOX (CO15). Time had a significant effect on K content in WSs from CB, CO15, and CO30, with slight and progressive enrichment between 60 and 270 days, likely released by the wood [38]. Steady concentrations were noticed for K during the last period of ageing (270–365 days). Although not significant, similar behaviour was observed for the CO60 and CN modalities of WSs. These levels of K are much lower that those found in ethanolic extracts prepared with different kinds of wood [40], suggesting low release of K from chestnut wood in comparison with oak, cherry, plum, and mulberry. Furthermore, higher concentrations of K are reported in the literature for WSs and other spirits aged with oak wood [5,8,10,43,56].

The results for Ca are of special technological interest, as this element may form insoluble compounds in WS, affecting negatively the WS clarity [10]. Thus, low concentrations of this element are advantageous from the technological perspective. No significant differences were observed between the ageing modalities (Table 1). Some variation in the content of this alkaline earth metal in the studied WSs over time was found. Strong enrichments occurred between 21 and 180 days (four- to eightfold), followed by significant depletions between 180 and 270 days, and finally (270–365 days) by a state characterized by an increasing tendency. Similar behaviour was noticed for WS from the N modality, despite no significant effect of the ageing time. Lowest and highest concentrations were shown by the wine distillate (not detected) and the WS from CO60 at 180 days (4.67 mg/L), respectively. The increase in concentration between 60 and 180 days may be explained by the delay in Ca release from the wood. WSs submitted to the highest level of MOX were expected to present higher amonts of CaO, which promptly reacts to form calcium hydroxide:

CaO (s) + H_2_O (l) ⇌ Ca(OH)_2_ (aq) (ΔH_r_ = −63.7 kJ/mol of CaO) (1)

As it hydrates, an exothermic reaction occurs which may also promote other changes to the reaction system. Another reasonable explanation for Ca depletion may be its precipitation as calcium tartrate (CaC₄H₄O₆), favoured by the high alcoholic strength of the WSs [53], despite the limited availability of tartaric acid in WSs in comparison with wine (solubility in water: 0.037 g/100 mLat 0 °C; 0.2 g/100 mL at 85 °C). With other ions competing for salt formation, Ca(OH)_2_ formation can also explain its variation over time (solubility in water: 1.73 g/L at 20 °C (retrograde solubility, i.e., decreasing with T)). Malic acid has been demonstrated to inhibit the velocity of CaC₄H₄O₆ crystallization [57]. The effect of various organic acids, including malic acid, on the precipitation of CaT was investigated in a hydroalcoholic model solution. McKinnon et al. [57] conducted these investigations and identified the following inhibitory order for each of the additives tested: citric acid > malic acid > lactic acid > succinic acid. These findings show that inhibitors with carboxyl groups or carboxyl and hydroxyl groups were the most efficient in inhibiting calcium tartrate.

Furthermore, the Ca concentrations found herein are in agreement with those reported for Sherry and Penedés spirits [8,18], tequila and mezcal spirits [11], cachaça, and cider spirits [43], and do not suggest a significant effect of the wood species [40].

The ageing technology impacted the level of Rb in WSs at 21 and 60 days of ageing (Table 1). For these ageing stages, significantly higher concentration was observed in the WS aged in barrel (643 ng/L, 365 days) in comparison with all the alternative ageing modalities, but was much lower that those found in whisk(e)y samples [12]. A general trend of enrichment over time, especially between 60 and 270 days, was observed in all the WSs. As for the Li and K elements, a tendency to decrease in the last stage of the ageing essay was observed.

Similar behaviour was observed for Sr, with significant effect of the ageing modality (Table 1). At each sampling time, the WS aged by the traditional technology (barrel) exhibited impressive higher concentration than those aged through the alternative modalities. The concentrations varied from 0.051 µg/L (CN, 60 days) to 5.177 µg/L (CB, 365 days). Time significantly affected Sr concentration, with progressive increases between 60 and 270 days, followed by decreases in the last stage of the ageing essay, except for the WS from CO15. Of particular interest was the sharp enrichment in Sr content in the first 21 days after putting the wine distillate in the wood barrels. For all the ageing modalities, the concentrations of Sr were much lower than those found in extraction solutions of other wood species [40], and much higher than those found in tequila and mezcal spirits [5,11,12].

In general, the most impressive increases of concentration were noticed between 60 and 270 days. From 270 to the end of the ageing experiment, and with exception of Na and Mg, concentrations seem to stabilize or even decrease, likely by insolubilization and precipitation phenomena. Finally, it is worth noting that for Rb and Sr, higher concentrations were found in WSs aged in barrels, as a consequence of the different balance between additive and subtractive phenomena, in comparison with MOX modalities. Hence, in future studies, this topic should be addressed.

#### 3.1.3. Heavy Metals and Other Elements

The occurrence of Al, Mn, Co, Ni, Zn, As, Cd, Mo, and Pb, most of them heavy metals, and their relationship with ageing technology and time, are reported in Table 2. For the Al element at 60 days, Cd and Mo at 30 days, and Pb at 365 days, data is not available; however, it is possible to foresee the evolution over time from the curve established with the measurements corresponding to five sampling dates. A significant effect of the ageing time was observed for all the metals, if not always in all the ageing modalities. This is most probably explained by the very low concentrations (consequently, with low precision associated to the measurements), and to the natural variability both of barrels and of staves. For several elements present in very low concentrations in the wine distillate, such as Al, Mn, Co, As, and Cd, slight enrichments occurred during ageing, suggesting transference from the wood, which is in accordance to the findings of [40] with several kinds of wood other than chestnut. In contrast, strong decreases were observed for Ni, Mo, and Pb, probably in accordance with the precipitation phenomena of heavy metals as insoluble salts, namely, as sulphides [38]. In fact, the use of sulphur dioxide (SO_2_) for the sanitation of wooden barrels and other containers is a common practice, and can lead to the presence of S in distilled spirits [11]. The depletion of these metals is quite positive from the quality perspective, given their potential toxicity.

Regarding Al, it combines characteristics of pre- and post-transition metals; however, it has no known function in living cells. Significant increases occurred in all WSs during ageing, with the lowest concentration being exhibited by the wine distillate (5.498 µg/L) and the highest one by the WS from CO15 at 365 days (57.304 µg/L) (Table 2). These levels are similar or lower than those reported in the literature for different spirits (brandy, whisk(e)y, sugarcane spirit, and tequila) [5,8,10,11,18,58]. For each sampling time, with the exception of 270 days, the Al content of the WSs was not significantly influenced by the ageing modality. However, the WS CO15 consistently showed the highest values, probably due to contamination of this modality at the beginning of the experiment. In fact, contamination with Al (the most abundant metal and the third most widespread element in the earth’s crust) is difficult to control during WS processing [59]. As for other metals, Al release from oak wood to spirit model is reported in the literature [40]. Al^3+^ occurs in aqueous solution as the hexaaqua cation [Al(H_2_O)_6_]^3+^. It may contribute to the acidity of the beverage since this cation can act as a proton donor and progressively hydrolyse until an Al(OH)_3_ precipitate forms. When the pH is raised, the hydroxide dissolves again, producing aluminate, [Al(H_2_O)_2_(OH)_4_]. Al monitoring is important because of its toxicity (e.g., neurodegenerative disorders) and because it can participate in haze formation (wines) and contribute to bitterness [4].

Progressive increases of Mn amounts were observed in all the ageing modalities over time, with the lowest concentration being found in the distillate (1.452 µg/L) and the highest one in the WS from CO15 modality, at 365 days (52.280 µg/L) (Table 2). No significant differences were noticed between ageing modalities at the end of the experiment. Presumably, enrichments of this essential element resulted from its release by the chestnut wood, as suggested by [40]. As aforementioned, it is thought that Mn^2+^ activates molecular oxygen by forming reactive oxygen species (e.g., hydroxyl radicals), similarly to Fe^3+^, probably catalysing oxidation reactions in WS. The contents of Mn in the studied WSs are comparable or lower than those reported for other spirits, namely, Serbian plum brandies [4], tequila and mezcal [11,60], whisk(e)y [12], grape spirits [21], several Transylvanian spirits [14], Brazilian sugarcane spirit [56], and Cognac WS [43].

Co is simultaneously an essential and a toxic metal, depending on the levels. Co content in the wine distillate (149 ng/L) seems to reflect transference from the distillation devices (Table 2). After a first stage of depletion, very slight enrichment, compatible with Smailagić et al. [4] observations for some botanical species, occurred between 60 and 270 days, with WS from CO15 showing the highest level (253 ng/L). Our results are of the same order of magnitude or lower than the reported levels for different spirits [22], including tequila [60], whisk(e)y [12], Transylvanian spirits [14], and cachaça [56]. Concerning the ageing technology, the distinct behaviour of CB and MOX modalities from 180 to 365 days was interesting: the former showed almost steady concentration, and the latter presented significant increases (180–270 days) followed by a decrease (270–365 days). Although there were no significant differences, the highest concentration was found in the CO15 modality, as observed for Al and Mn elements.

Ni, which is also an essential and toxic element, showed similar kinetics to Co during the ageing experiment. In this case, the wine distillate exhibited the highest concentration (5.393 µg/L), likely due to the contact with metallic surfaces (Table 2). Drastic depletion occurred over time: the final concentrations (at 365 days) were always below 2 µg/L, similar to those reported for tequila [60] and lower than whisk(e)y [12]. At the end of the trial, no significant differences were detected between the ageing technologies and between the alternative modalities; however, CB and CO15 displayed the highest values.

For Zn, although no significant effect of ageing technology was observed, concentrations tend to be higher in WSs from CB. It must be emphasized that the behaviour exhibited by the two barrels (independent replicates), in respect to this essential metal, was quite dissimilar (Table 2), explaining the lack of significance of the statistical test. These results highlight the natural variation between similar barrels (made in the same cooperage, using the same kind of wood, from the same geographical origin), which was recently examined by [61]. The results for CB suggest a strong release of Zn at the beginning of the trial, counterbalancing any possible loss by the precipitation phenomenon, as those evidenced for WSs from the MOX modalities. With the exception of WSs from CB (256 µg/L), the highest concentration was found in the wine distillate (86 µg/L). Compared with other spirits, Zn concentrations in the studied WSs were of the same magnitude of those reported for tequila, mezcal, and whiskey [11,12,60], and were lower than those found in plum spirits aged in oak barrels [4]. The results obtained comply with the standard limit set by the International Organisation of Vine and Wine (OIV) of 2 mg/L for wine [62], which is equal to the limit established in Serbian regulations for fruit spirits [4]. Zn might be an attractive transition metal to study, not only for its biological qualities [63], but also for its catalytic abilities [64], which could play a part in the dynamic ageing processes of WSs.

With regard to As, a highly toxic element that is notably carcinogenic, a slight and progressive enrichment over time occurred, suggesting external contamination of CO30, CO60, and CN samples at the end of the experiment (365 days) (Table 2). Excluding this sampling time, WSs from CB tend to present higher concentrations than the others, pointing to a more intense extraction of this mineral element from the wooden barrel. These observations contradict the results of Smailagić et al. [40] study that revealed no evidence of As extraction from different kinds of wood to spirit model solution. It should be emphasized that, excluding the WSs from CO30, CO60, and CN (365 days), the concentrations were always extremely low (<0.4 µg/L), compared with tequila and mezcal [60], and especially with Transylvanian spirits (from several fruits’ distillates) in which levels from 4 to 50 µg/L were found [14]. It is noteworthy that OIV establishes the maximum acceptable limit of 0.200 mg/L for wines [62,65], very far (500-fold) from the levels found in the WSs of this study, especially keeping in mind that the daily intake of WS is much lower than that of wine.

Time had significant effect on Cd levels, with slight enrichments until 180 days of ageing, followed by depletion until 270 days (Table 2). After that, slight variations were observed, except for the WSs from CN. This behaviour probably resulted from the release of this toxic metal from the wood, achieving a concentration exceeding solubility and leading to precipitation. The decrease of pH over time, mainly due to the increase of acetic acid [42], may have been another critical factor, favouring Cd solubility (soluble in water, methanol and ethanol (anhydrous), very soluble (dihydrate)), thus contributing to the explanation of the evolution between 270 and 365 days. In aqueous solution, acetic acid undergoes ionization and further coordination with Cd, according to the Equations (2) and (3):

CH_3_COOH (aq) + H_2_O (l) ⇌ H_3_O^+^ (aq) + CH_3_CO_2_^−^ (aq) (2)

CdO (s) + 2 CH_3_CO_2_H (aq) + H_2_O (l) → Cd(O_2_CCH_3_)_2_(H_2_O)_2_ (aq) (3)

A reasonable explanation for the results attained for the WSs from CN, distinct from the others, might be the low levels of dissolved oxygen in these spirits (Figure 2), influencing the oxidation-reduction potential of the medium, consequently, the oxidation forms of Cd, and, finally, its solubility. As aforementioned, the literature lacks information on mineral release from chestnut wood, though data for Cd extraction from wood of other botanical species is available, apparently without significant effect of the species [40]. Furthermore, the concentrations measured in the WSs at 365 days, around 100 ng/L, are very close to those reported for tequila [60] and lower than the values reported for whisk(e)y [12] and different kinds of Transylvanian spirits [14]. The maximum acceptable limit for this metal in wine is of 0.01 mg/L [62].

Mo and Pb exhibited similar behaviour, with the highest concentrations in the wine distillate, of 246 ng/L and 8.024 µg/L, respectively, most probably introduced during the distillation process (Table 2). For both elements, sharp depletions occurred until 60 days of ageing, likely by insolubilisation and precipitation phenomena. Therefore, due to very low concentration of Pb in the samples, no concentration values were retained for 365 days. Nevertheless, it can be assumed that between 270 and 365 days, the concentrations of Pb continued to decrease. In particular, the Pb depletion (> 90%) is extremely positive considering the high toxicity of this metal. Presumably, the amounts of Mo and Pb extracted from wood to WSs have not been high enough to counterbalance their losses by precipitation. At the end of the essay, the highest content of Mo was found in the CO30 modality (83 ng/L), with CO15 showing the highest level of Pb (0.540 µg/L). Mo is a well-known catalyst used for both reduction and oxidation processes due to its various coordinative geometries offered by the most distinct oxidation states, extending from 2+ to 6+. Several catalytic examples of high-valence oxomolybdenum complexes have proven to be effective catalysts for the reduction of organic molecules, some of which are related to WS substrates, such as aldehydes and ketones [66], and esters [67]. At the end of the essay, no significant effect of the ageing technology/modality of ageing on Mo and Pb content was noted. It is worth mentioning that currently, the maximum acceptable limit for Pb in wine is 0.150 mg/L [62,68]. Within Serbian regulations, the maximum concentration of 0.5 mg/L in fruit spirits has been established [4]. In addition, the levels of Mo in these WSs are much lower than those reported for tequila [60], whisk(e)y [12], and diverse Transylvanian spirits [14]. In addition, the concentrations of Pb found in the WSs under study were lower than those found in Spanish spirits [18], tequila [60], and other spirits [10,14,21].

Lastly, As, Cd, and Pb (in addition to Hg, which is almost absent in wine spirits, as contamination sources are very unlikely), being highly toxic and/or carcinogenic metals, are the most notorious among heavy metals [69]. All of these metals have the potential to react with biological systems by losing one or more electrons and generating metal cations with affinity for the nucleophilic sites of essential macromolecules. Heavy metals have a variety of acute and chronic harmful effects on various human organs, and the exposure to two or more metals at the same time may have cumulative effects [17]. Surprisingly, no maximum acceptable limits are currently established for these or any other metals in WS by the OIV. Bearing in mind that the consumption patterns of wine and WS are quite different, the thresholds defined for wine are only indicative. The increasing concerns regarding health effects and environmental exposure will probably result in the monitoring of potentially toxic elements in WS, in a near future, since exposure to these metals has been increased by both industrial and anthropogenic activities.

### 3.2. Global Behaviour of Mineral Elements

To complement the individual approach based on the ANOVA results, a global analysis was performed using cluster heat maps to understand the behaviour of the mineral elements as a whole, and particularly to examine if they could be helpful for discriminating the WSs corresponding to the different technologies and ageing modalities.

**Figure 4:**
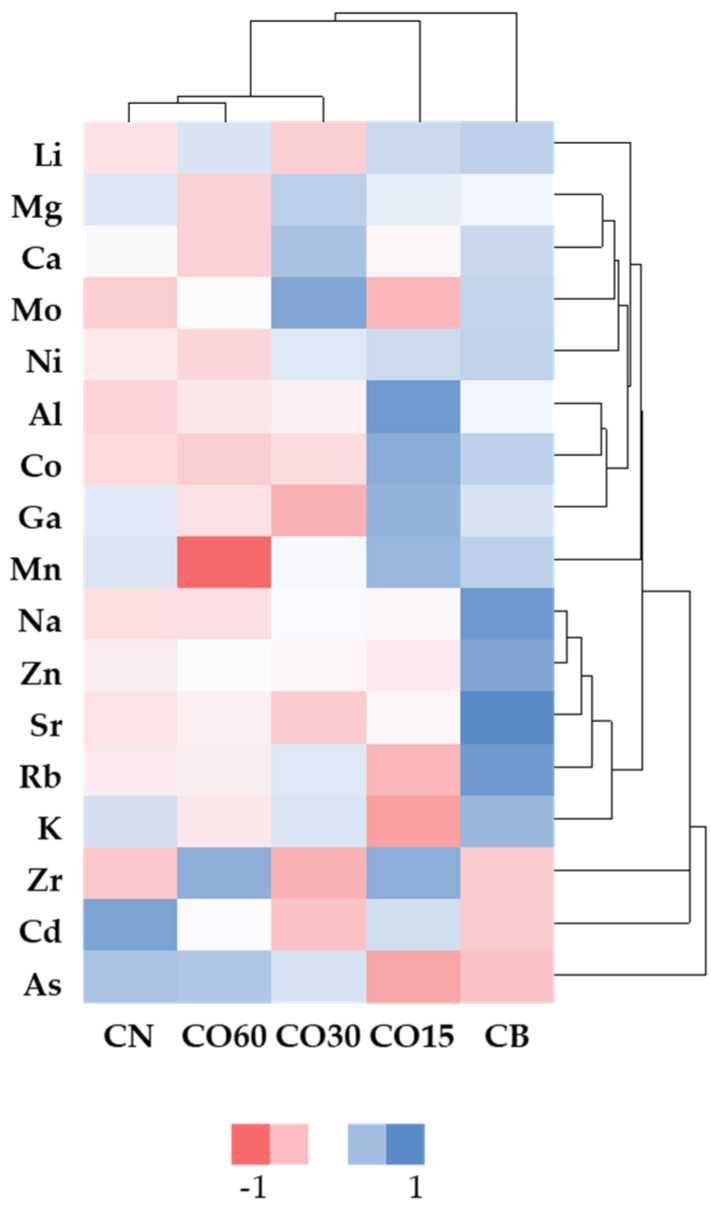
Heat map plotting clusters of mineral elements and ageing modalities of wine spirits at 365 days of ageing.

The heat map displayed in Figure 4 was generated from the content of the mineral elements previously discussed, plus Ga and Zr concentrations, in WSs at the end of the ageing experiment (365 days). Blue colours represent a positive correlation between the analytes’ levels and the ageing modalities while pink colours depict a negative correlation between them.

The heat map clustered the WSs into two groups according to the ageing technology: the first group comprised the barrel (CB) and the second group contained the ageing modalities from the MOX technology and the control one. This result evidences the different balance between additive and subtractive phenomena in barrel in comparison with MOX modalities, probably explained by distinct oxidative media. WSs from CB are positively correlated with most of the elements, in particular with Na, K, Zn, Rb, and Sr, and negatively correlated with As, Zr, and Cd. Regarding MOX modalities, CO15 stands out from the rest, being the most similar to the barrel. Al, Mn, Co, Ga, and Zr are positively correlated with this MOX modality. The other MOX modalities are included in the same subcluster, with CN and O60 exhibiting a weak negative correlation with most of the elements. Excluding As, for the aforementioned reasons, the heat map clustered the mineral elements into three major groups: the first one contains Cd; the second one corresponds to Zr; the last one comprises the other elements. It is also possible to identify several subclusters without a clear distinction between them. Finally, these observations corroborate the ANOVA ones.

## 4. Conclusions

This study provides innovative information on the effect of chestnut wood on WS mineral composition, considering both traditional ageing (wooden barrels) and alternative technology (staves combined with different micro-oxygenation levels). With the exception of Sr, there were no significant differences between WSs from different ageing modalities at the conclusion of the ageing essay (365 days). Yet, the WSs aged in barrels tend to present higher concentrations of Na, K, Rb, and Zn, in addition to Sr. The results evidenced barrel-to-barrel variation as regards the extraction of mineral elements to wine spirits. Ageing time had significant effect on most of the metals, with different trends and a distinct magnitude of changes, depending on the element. In general, enrichments of alkaline and alkaline earth metals occurred over time, together with strong depletions of Ni, Mo, and Pb. In future studies, the different physicochemical forms of the mineral elements (speciation analysis) that together constitute the total concentrations, as well as the balance of their different oxidation forms, should be assessed for a better understanding of their kinetics, risk of haze formation, and potential toxicity, among others. After 365 days of ageing, the most abundant metals in WSs were Ca, Mg, and Na (1–2 mg/L), followed by Cu and Fe [3]. The concentrations of mineral elements, especially those of heavy metals, found in the WSs of this study were very low—of the same order of magnitude or lower than those reported for other spirits, which is quite positive from the quality and safety points of view. Lastly, these findings reinforce the appropriateness of chestnut wood for the ageing of wine spirits.

## Figures and Tables

**Table 1:** Concentration of alkaline and alkaline earth elements quantified in the WSs sampled from different ageing modalities over time, and ANOVA summary.

			Time (Days)						
	Modality		0	21	60	180	270	365	
**Li (µg/L)**	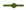	**CB**	1.40 ± 0.05	1.2 ± 0.3	1.5 ± 0.3	2.5 ± 0.8	3 ± 1	3.2 ± 0.8	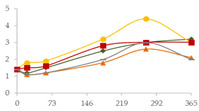
	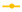	**O15**	1.40 ± 0.05 *a*	1.8 ± 0.2 *a*	1.9 ± 0.5 *a*	3.2 ± 0.4 *b*	4.4 ± 0.2 *c*	3.0 ± 0.5 *b*	
	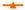	**O30**	1.40 ± 0.05 *a*	1.1 ± 0.3 *a*	1.2 ± 0.1 *a*	1.8 ± 0.2 *b*	2.6 ± 0.1 *c*	2.1 ± 0.3 *b*	
	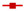	**C60**	1.40 ± 0.05	1.5 ± 0.9	1.6 ± 0.8	3 ± 1	3 ± 2	3 ± 2	
	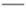	**N**	1.40 ± 0.05	1.1 ± 0.5	1.2 ± 0.5	2 ± 1	2 ± 1	2 ± 1	
**Na (µg/L)**	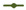	**CB**	698 ± 51 *ab*	611 ± 6 *a C*	406 ± 143 *a*	920 ± 154 *abc*	1249 ± 154 *bc*	1459 ± 371 *c*	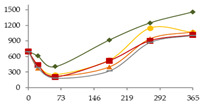
	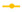	**O15**	698 ± 51 *c*	425 ± 5 *ab B*	249 ± 72 *a*	529 ± 7 *bc*	1146 ± 200 *d*	1061 ± 123 *d*	
	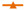	**O30**	698 ± 51 *b*	379 ± 11 *a A*	222 ± 59 *a*	400 ± 41 *a*	943 ± 159 *bc*	1075 ± 13 *c*	
	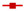	**C60**	698 ± 51 *bc*	437 ± 9 *ab B*	207 ± 20 *a*	519 ± 19 *b*	903 ± 254 *cd*	1013 ± 38 *d*	
	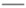	**N**	698 ± 51 *b*	392 ± *10 a A*	185 ± 69 *a*	324 ± 39 *a*	872 ± 235 *bc*	1012 ± 69 *c*	
**Mg (µg/L)**	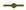	**CB**	128 ± 11 *a*	603 ± 133 *bc C*	389 ± 3 *ab B*	799 ± 42 *cd*	1017 ± 147 *d*	1413 ± 248 *e*	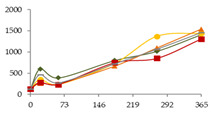
	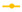	**O15**	128 ± 11 *a*	351 ± 63 *a AB*	234 ± 2 *a A*	731 ± 122 *b*	1368 ± 254 *c*	1440 ± 99 *c*	
	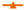	**O30**	128 ± 11 *a*	264 ± 79 *a A*	248 ± 8 *a A*	668 ± 112 *b*	1097 ± 16 *c*	1544 ± 374 *d*	
	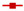	**C60**	128 ± 11 *a*	286 ± 19 *a AB*	235 ± 19 *a A*	749 ± 242 *b*	852 ± 306 *b*	1310 ± 202 *c*	
	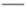	**N**	128 ± 11 *a*	470 ± 16 *ab BC*	275 ± 83 *ab A*	724 ± 70 *bc*	1069 ± 413 *cd*	1464 ± 252 *d*	
**K (µg/L)**	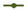	**CB**	6 ± 1 *a*	306 ± 110 *bc*	173 ± 26 *ab*	420 ± 99 *bc*	524 ± 166 *c*	506 ± 109 *c*	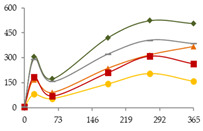
	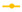	**O15**	6 ± 1 *a*	81 ± 3 *bc*	56 ± 27 *ab*	144 ± 19 *cd*	206 ± 28 *d*	159 ± 48 *d*	
	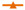	**O30**	6 ± 1 *a*	167 ± 54 *abc*	92 ± 51 *ab*	236 ± 111 *bcd*	317 ± 137 *cd*	367 ± 17 *d*	
	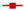	**C60**	6 ± 1	185 ± 148	71 ± 55	211 ± 154	310 ± 158	264 ± 142	
	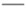	**N**	6 ± 1	292 ± 299	158 ± 157	322 ± 258	406 ± 314	386 ± 303	
**Ca (µg/L)**	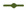	**CB**	nd	433 ± 195 *a*	936 ± 96 *ab*	4218 ± 5 *c*	1517 ± 478 *ab*	1966 ± 1122 *b*	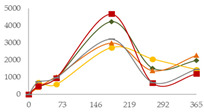
	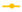	**O15**	nd	677 ± 76 *a*	572 ± 53 *a*	2714 ± 154 *b*	2053 ± 241 *b*	1438 ± 1070 *ab*	
	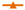	**O30**	nd	405 ± 33 *a*	1031 ± 306 *ab*	2985 ± 38 *c*	1372 ± 594 *b*	2262 ± 48 *c*	
	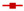	**C60**	nd	490 ± 117 *a*	941 ± 426 *a*	4674 ± 16 *b*	652 ± 568 *a*	1198 ± 1099 *a*	
	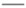	**N**	nd	732 ± 675	964 ± 361	3216 ± 1973	695 ± 821	1464 ± 302	
**Rb (ng/L)**	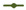	**CB**	151 ± 28 *a*	433 ± 146 *bc B*	352 ± 41 *abB*	507 ± 116 *bcd*	689 ± 116 *d*	643 ± 114 *cd*	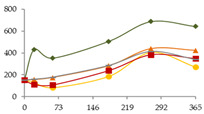
	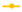	**O15**	151 ± 28 *a b*	128 ± 15 *ab A*	81 ± 20 *a A*	185 ± 23 *ab*	405 ± 76 *c*	270 ± 143 *bc*	
	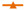	**O30**	151 ± 28	156 ± 20 *A*	175 ± 90 *A*	283 ± 81	440 ± 123	424 ± 139	
	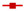	**C60**	151 ± 28 *a*	111 ± 13 *a A*	106 ± 34 *a A*	238 ± 131 *ab*	383 ± 71 *b*	348 ± 93 *b*	
	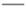	**N**	151 ± 28	160 ± 44 *A*	178 ± 47 *A*	286 ± 129	416 ± 214	342 ± 59	
**Sr (ng/L)**	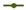	**CB**	544 ± 40 *a*	3531 ± 680 *bc B*	2586 ± 170 *b B*	3607 ± 108 *bc B*	5177 ± 1416 *c B*	3726 ± 739 *bc B*	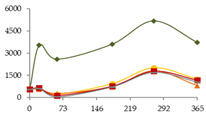
	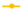	**O15**	544 ± 40	623 ± 119 *A*	218 ± 153 *A*	928 ± 127 *A*	2006 ± 929 *A*	1238 ± 820 *A*	
	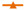	**O30**	544 ± 40 *a*	549 ± 107 *a A*	259 ± 12 *a A*	725 ± 222 *aA*	1802 ± 376 *b A*	804 ± 343 *a A*	
	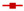	**C60**	544 ± 40 *ab*	645 ± 116 *b A*	139 ± 46 *a A*	765 ± 144 *bc A*	1777 ± 63 *d A*	1169 ± 409 *c A*	
	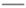	**N**	544 ± 40 *b*	609 ± 190 *bc A*	52 ± 25 *a A*	720 ± 48 *bc A*	1688 ± 320 *d A*	1048 ± 249 *c A*	

Results expressed as mean ± standard deviation. For each compound: ageing time (days)—means within the same row followed by different lowercase letters (*a,b,c*) are significantly different (*p* < 0.05); ageing modality—means within the same column followed by different uppercase letters (*A,B,C*) are significantly different (*p* < 0.05); nd—not detected; 0 days—corresponds to the wine distillate used to fill the demijohns; CB—barrel; CO15, CO30, and CO60—MOX levels; CN—Nitrogen (control).

**Table 2:** Concentration of heavy metals and other elements quantified in the WSs sampled from different ageing modalities over time, and ANOVA summary.

			Time (Days)						
	Modality		0	21	60	180	270	365	
**Al (µg/L)**	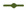	**CB**	5.5 ± 0.8 *a*	8 ± 2 *a*	-	21 ± 2 *b*	24 ± 3 *b A*	32 ± 2 *c*	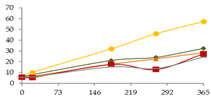
	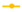	**O15**	5.5 ± 0.8	10 ± 2	-	32 ± 7	46 ± 14 *B*	57 ± 27	
	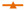	**O30**	5.5 ± 0.8 *a*	6 ± 3 *a*	-	18 ± 3 *b*	22.7 ± 0.5 *bc A*	28 ± 4 *c*	
	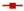	**C60**	5.5 ± 0.8 *a*	5.3 ± 0.5 *a*	-	18 ± 1 *b*	13 ± 1 *b A*	27 ± 4 *c*	
	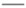	**N**	5.5 ± 0.8 *a*	6.0 ± 0.3 *a*	-	15 ± 4 *ab*	14 ± 5 *ab A*	25 ± 8 *b*	
**Mn (µg/L)**	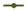	**CB**	1.5 ± 0.1 *a*	13 ± 1 *b C*	18 ± 2 *b D*	37 ± 3 *c*	42 ± 4 *cd*	47 ± 4 *d*	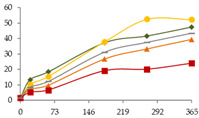
	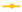	**O15**	1.5 ± 0.1 *a*	10 ± 1 *ab BC*	15.4 ± 0.2 *b CD*	38 ± 6 *c*	52 ± 9 *d*	52 ± 5 *d*	
	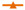	**O30**	1.5 ± 0.1 *a*	7 ± 2 *a AB*	10 ± 2 *a AB*	27 ± 6 *b*	33 ± 6 *b*	39 ± 13 *b*	
	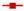	**C60**	1.5 ± 0.1 *a*	5 ± 2 *a A*	7 ± 2 *a A*	19 ± 6 *b*	20 ± 2 *b*	24 ± 5 *b*	
	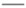	**N**	1.5 ± 0.1 *a*	8.4 ± 0.1 *b AB*	12.2 ± 0.6 *b BC*	31 ± 5 *c*	37 ± 2 *d*	43 ± 1 *e*	
**Co (ng/L)**	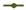	**CB**	149 ± 7 *c*	23 ± 30 *a*	64 ± 11 *ab BC*	122 ± 15 *bc B*	153 ± 43 *c A*	149 ± 20 *c*	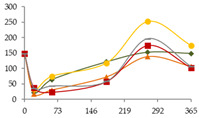
	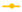	**O15**	149 ± 7 *bc*	34 ± 15 *a*	75 ± 20 *a C*	118 ± 22 *b B*	253 ± 20 *d B*	174 ± 12 *c*	
	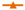	**O30**	149 ± 7 *c*	18 ± 14 *a*	30 ± 4 *a A*	73 ± 1 *ab A*	139.0 ± 0.3 *c A*	107 ± 63 *bc*	
	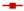	**C60**	149 ± 7 *c*	38 ± 5 *a*	23 ± 10 *a A*	58 ± 24 *a A*	174 ± 21 *c A*	102 ± 25 *b*	
	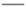	**N**	149 ± 7 *bc*	37 ± 20 *a*	44 ± 6 *a AB*	58.4 ± 0.1 *a A*	197 ± 15 *c AB*	107 ± 33 *b*	
**Ni (µg/L)**	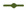	**CB**	5.4 ± 0.3 *d*	2.323 ± 0.004 *bc*	1.49 ± 0.08 *a B*	1.49 ± 0.08 *a B*	2.6 ± 0.2 *c*	1.6 ± 0.5 *ab*	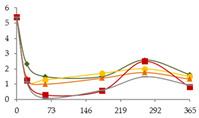
	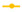	**O15**	5.4 ± 0.3 *c*	1.25 ± 0.06 *a*	1.3 ± 0.2 *b B*	1.70 ± 0.06 *ab B*	2.0 ± 0.4 *ab*	1.5 ± 0.4 *a*	
	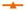	**O30**	5.4 ± 0.3 *b*	1.39 ± 0.06 *a*	1.012 *a B*	1.39 ± 0.01 *a B*	1.8 ± 0.7 *a*	1.3 ± 1.5 *a*	
	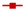	**C60**	5.4 ± 0.3 *b*	1 ± 1 *a*	0.289 *a A*	0.5 ± 0.2 *a A*	2.5 ± 0.9 *a*	0.8 ± 0.5 *a*	
	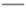	**N**	5.4 ± 0.3 *c*	1.2 ± 0.6 *ab*	0.088±0.088 *a A*	0.61 ± 0.02 *ab A*	1.5 ± 0.2 *b*	0.9 ± 1.0 *ab*	
**Zn (µg/L)**	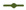	**CB**	86 ± 3	229 ± 262	137 ± 161	224 ± 234	218 ± 247	256 ± 311	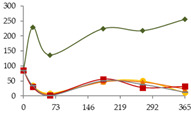
	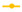	**O15**	86 ± 3 *c*	35 ± 7 *b*	9 ± 11 *a*	48 ± 0.8 *b*	50 ± 7 *b*	11 ± 4 *a*	
	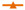	**O30**	86 ± 3 *e*	32 ± 1 *bc*	6 ± 2 *a*	47 ± 10 *d*	46 ± 6 *cd*	23 ± 9 *b*	
	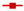	**C60**	86 ± 3 *d*	30 ± 2 *b*	2.0 *a*	56 ± 9 *c*	27.8 ± 0.8 *b*	31.5 ± 0.6 *b*	
	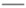	**N**	86 ± 3 *c*	35 ± 3 *b*	1.2 ± 0.3 *a*	51 ± 15 *b*	38 ± 2 *b*	12 ± 3 a	
**As (ng/L)**	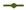	**CB**	26 ± 4 *a*	188 ± 35 *b*	187 ± 41 *b B*	317 ± 14 *c*	232 ± 15 *b D*	353 ± 38 *c*	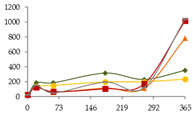
	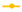	**O15**	26 ± 4 *a*	138 ± 21 *b*	152 ± 41 *b B*	196 ± 39 *bc*	201 ± 4 *bc CD*	234 ± 48 *c*	
	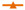	**O30**	26 ± 4 *a*	132 ± 29 *c*	60 ± 17 *ab A*	117 ± 8 *bc*	113 ± 18 *bc A*	786 ± 44 *d*	
	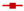	**C60**	26 ± 4 *a*	126 ± 59 *ab*	67 ± 20 *ab A*	107 ± 4 *ab*	172 ± 29 *b BC*	1020 ± 106 *c*	
	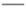	**N**	26 ± 4	155 ± 27	53 ± 7 *A*	198 ± 133	126 ± 22 *AB*	1041 ± 872	
**Cd (ng/L)**	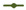	**CB**	7.94 *a*	-	38 ± 13 *a*	312 ± 33 *c B*	107 ± 12 *b*	78 ± 23 *ab*	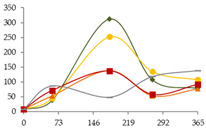
	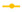	**O15**	7.94	-	44 ± 17	252 ± 38 *B*	135 ± 101	107	
	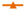	**O30**	7.94 *a*	-	53 ± 16 *ab*	138 ± 12 *cA*	53 ± 14 *ab*	76.6 *b*	
	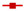	**C60**	7.94	-	71 ± 12	137 ± 62 *A*	58 ± 11	92 ± 39	
	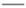	**N**	7.94	-	86 ± 92	48 ± 23 *A*	118 ± 30	138 ± 28	
**Mo (ng/L)**	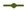	**CB**	246 ± 18 *c*	-	54 ± 3 *a D*	64 ± 10 *a*	102 ± 4 *b*	62 ± 5 *a*	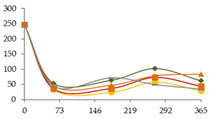
	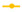	**O15**	246 ± 18 *c*	-	34 ± 3 *ab A*	24 ± 8 *a*	58 ± 8 *ab*	28 ± 1 *ab*	
	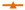	**O30**	246 ± 18 *c*	-	46 ± 4 *a CD*	47 ± 12 *a*	78 ± 10 *a*	83 ± 4 *ab*	
	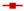	**C60**	246 ± 18 *b*	-	36 ± 4 *a AB*	36 ± 22 *a*	73 ± 20 *a*	44 ± 20 *a*	
	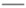	**N**	246 ± 18 *b*	-	43 ± 2 *a BC*	71 ± 41 *a*	49 ± 25 *a*	34 ± 39 *a*	
**Pb (ng/L)**	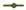	**CB**	8025 ± 181 *c*	1654 ± 155 *b B*	583 ± 100 *a*	817 ± 76 *a*	462 *a*	-	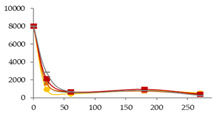
	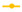	**O15**	8025 ± 181 *b*	931 ± 44 *a A*	447 ± 172 *a*	804 ± 400 *a*	540 ± 287 *a*	-	
	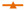	**O30**	8025 ± 181 *d*	1628 ± 111 *c B*	689 ± 64 *ab*	755.3 ± 0.4 *b*	370 ± 137 *a*	-	
	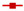	**C60**	8025 ± 181 *c*	2156 ± 5 *b C*	663 ± 500 *a*	956 ± 63 *a*	407 ± 52 *a*	-	
	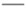	**N**	8025 ± 181 *d*	2879 ± 213 *c D*	686 ± 167 *ab*	829 ± 239 *b*	273 ± 10 *a*	-	

Results expressed as mean ± standard deviation. For each compound: ageing time (days)—means within the same row followed by different lowercase letters (*a,b,c*) are significantly different (*p* < 0.05); ageing modality—means within the same column followed by different uppercase letters (*A,B,C*) are significantly different (*p* < 0.05); nd—not detected; 0 days—corresponds to the wine distillate used to fill the demijohns; CB—barrel; CO15, CO30, and CO60—MOX levels; CN—Nitrogen (control).

## Data Availability

Data are contained within the article.
